# Determination of Luteolin-7-O-diglucuronide in *Perilla frutescens* (L.) Britt. Leaf Extracts from Different Regions of China and Republic of Korea and Its Cholesterol-Lowering Effect

**DOI:** 10.3390/molecules28207007

**Published:** 2023-10-10

**Authors:** Zhaoyang Wu, Sangyoun Lee, Beomgoo Kang, Sookyeong Lee, Kyochul Koo, Jaeyong Lee, Soonsung Lim

**Affiliations:** 1 Department of Food Science and Nutrition, Hallym University, 1 Hallymdeahak-gil, Chuncheon 24252, Republic of Korea; wzy19970202@163.com (Z.W.); ilove0977@nate.com (S.L.); 2 Institute for Liver and Digestive Diseases, Hallym University, 1 Hallymdeahak-gil, Chuncheon 24252, Republic of Korea; briansylee@naver.com; 3 Department of Biochemistry, College of Medicine, Hallym University, 1 Hallymdeahak-gil, Chuncheon 24252, Republic of Korea; kbgda87@naver.com (B.K.); jyl3746@gmail.com (J.L.); 4 Institute of Korean Nutrition, Hallym University, 1 Hallymdeahak-gil, Chuncheon 24252, Republic of Korea; 5 COSFarm Co., Ltd., Corporate Research Institute, 3F 162, Saeteo-gil, Seonggeo-eup, Seobuk-gu, Cheonan-si 12446, Republic of Korea; rnrycjf76@naver.com

**Keywords:** *Perilla frutescens* (L.) Britt., Luteolin-7-O-diglucuronide, hypercholesterolemia cholesterol-lowering effect

## Abstract

Lowering blood cholesterol levels is crucial for reducing the risk of cardiovascular disease in patients with familial hypercholesterolemia. To develop *Perilla frutescens* (L.) Britt. leaves as a functional food with a cholesterol-lowering effect, in this study, we collected *P. frutescens* (L.) Britt. leaves from different regions of China and Republic of Korea. On the basis of the extraction yield (all components; g/kg), we selected *P. frutescens* (L.) Britt. leaves from Hebei Province, China with an extract yield of 60.9 g/kg. After evaluating different concentrations of ethanol/water solvent for *P. frutescens* (L.) Britt. leaves, with Luteolin-7-O-diglucuronide as the indicator component, we selected a 30% ethanol/water solvent with a high Luteolin-7-O-diglucuronide content of 0.548 mg/g in *Perilla. frutescens* (L.) Britt. leaves. Subsequently, we evaluated the cholesterol-lowering effects of *P. frutescens* (L.) Britt. leaf extract and Luteolin-7-O-diglucuronide by detecting total cholesterol in HepG2 cells. The 30% ethanol extract lowered cholesterol levels significantly by downregulating 3-hydroxy-3-methyl-glutaryl-coenzyme A reductase expression. This suggests that *P. frutescens* (L.) Britt leaves have significant health benefits and can be explored as a potentially promising food additive for the prevention of hypercholesterolemia-related diseases.

## 1. Introduction

Familial hypercholesterolemia is a genetic, lipid-related, monogenic, and autosomal dominant disorder [1]. It is characterized by elevated low-density lipoprotein cholesterol (LDL-C) levels, premature atherosclerotic cardiovascular disease (ASCVD), and high mortality. Hypercholesterolemia contributes to a higher risk of atherosclerotic cardiovascular disease than other causes of dyslipidemia at all LDL cholesterol levels [2]. Preventing ASCVD in individuals with hypercholesterolemia usually requires lifelong adherence to cholesterol-lowering therapies [3]. Biosynthesis of cholesterol in the liver is mainly controlled by sterol regulatory element-binding protein 2 in order to modulate the expression of HMG-CoA reductase, one of the main enzymes of cholesterol synthesis [4]. Therefore, controlling cholesterol metabolism by regulating the expression of HMG-CoA reductase is necessary to suppress secondary diseases (cardiovascular and cerebrovascular diseases) caused by high cholesterol.

Statins, among the most widely used drugs worldwide, reduce LDL-C by 30–50% on average [5]. Although statins are generally considered “very safe and well tolerated”, concerns have arisen regarding the management of certain patient groups owing to reports of muscular complications, increased risk of diabetes, and temporary elevation of liver aminotransferase levels. These concerns significantly affect patients’ quality of life, causing considerable inconvenience [6,7]. The utilization of natural medicines for the treatment of various diseases and disorders has a long history dating back to ancient times. Therefore, the prominent utilization of herbal drugs as a fundamental approach is essential for the management, prevention, and treatment of hypercholesterolemia. Therefore, there is an urgent need to discover bioactive substances from natural products that lower blood cholesterol while producing few side effects.

*Perilla frutescens* (L.) Britt., an annual herbal plant belonging to the mint family *Lamiaceae*, is extensively cultivated in various Asian countries, including China, Japan, South Korea, Vietnam, and India [8]. The traditional uses of *P. frutescens* (L.) Britt. include two aspects: culinary and medicinal uses. In terms of culinary uses, the utilization of *P. frutescens* (L.) Britt. leaves as a culinary aromatic for fish preparations has a historical trajectory spanning over 2000 years in China [9]. Furthermore, *P. frutescens* (L.) Britt. serves as a representative flavor in Japan and a spicy vegetable in Korea [10]. Additionally, the seeds of *P. frutescens* (L.) Britt. hold significant global importance as a primary reservoir of perilla oil, owing to their substantial omega-3 fatty acid content. In terms of medicinal uses, according to the Chinese Pharmacopeia 2015, different parts of *P. frutescens* (L.) Britt. have been utilized as natural herbal medicines to alleviate various symptoms. *P. frutescens* (L.) Britt. leaves are noted for their therapeutic attributes, including the capacity to disperse surface pathogenic factors, alleviate cold conditions, and promote gastric function. *P. frutescens* (L.) stems are recognized for their potential to promote qi circulation, alleviate pain, and assist in ensuring a safe pregnancy. Moreover, *P. frutescens* (L.) seeds are acknowledged for their efficacy in promoting qi circulation, resolving phlegm, alleviating coughs, easing respiratory distress, and facilitating intestinal regularity [11]. Contemporary famous doctors created multiple new prescriptions based on ancient classic prescriptions. On the basis of San-Zi-Yang-Qin decoction, Raphani semen, Sinapis semen, and *P. frutescens* (L.) Britt were added to a cure for nonalcoholic fatty liver disease [12]. Many studies have revealed the pharmacological properties of *P. frutescens* (L.) Britt., including its antioxidant, antibacterial, antifungal, antiallergic, antidepressant, anti-inflammatory, and antitumor effects [13,14,15,16,17]. Over 200 phytoconstituents have been isolated from *P. frutescens* (L.) Britt., including alkaloids, phenylpropanoids, terpenoids, polyphenolic compounds, and flavonoids [8]. Furthermore, studies have shown that the total flavonoid extract of *P. frutescens* mainly contains Luteolin-7-O-diglucuronide, caffeic acid, scutellarin, apigenin-7-glucuronide, and rosmarinic acid, which inhibited hyperlipidemia in rats fed with a high-fat diet [10,18,19,20]. Therefore, *P. frutescens* (L.) Britt. leaves are a promising source of new functional food ingredients to lower cholesterol levels and improve hypercholesterolemia treatments.

Therefore, in this study, the extraction yields (all components; %) of *P. frutescens* (L.) Britt. leaves from China and Republic of Korea were evaluated, using Luteolin-7-O-diglucuronide as an indicator component to establish a quantitative method for Luteolin-7-O-diglucuronide; the extraction solvent was selected on the basis of the content of Luteolin-7-O-diglucuronide (mg/g in *P. frutescens* (L.) Britt. leaves). The cholesterol-lowering effect of *P. frutescens* (L.) Britt. leaf extract and Luteolin-7-O-diglucuronide was studied by detecting total cholesterol in HepG2 cells. This study indicated that *P. frutescens* (L.) Britt. leaf extract had good activity in terms of its cholesterol-lowering effect, which provides a theoretical basis for the development of *P. frutescens* (L.) Britt. leaves as a functional food for their cholesterol-lowering effect.

## 2. Results and Discussion

### 2.1. Method Validation

**Figure 1:**
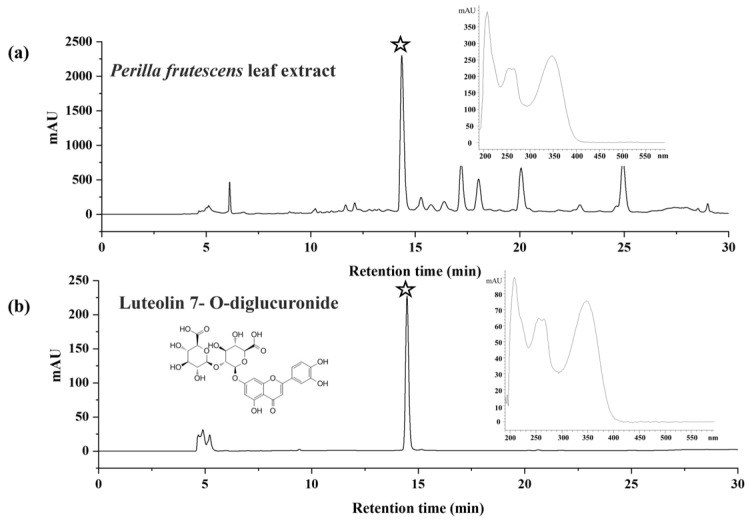
HPLC spectra of 30% ethanol extract of *P. frutescens* (L.) Britt. leaves from Hebei Province, China (**a**) and Luteolin-7-O-diglucuronide (**b**), stars in the figure are the target compound in the 30% ethanol extract of *P. frutescens* (L.) Britt. leaves.

**Figure 2:**
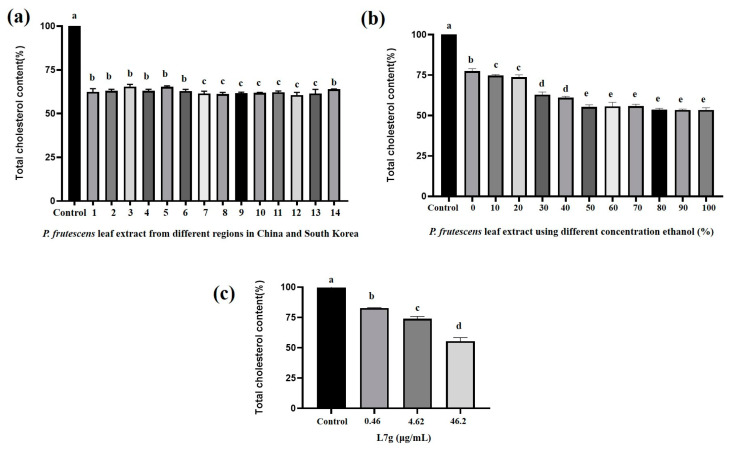
Cholesterol-lowering effects of *P. frutescens* (L.) Britt. leaf extract from different regions in China and Republic of Korea (**a**); *P. frutescens* (L.) Britt. leaf extract using different concentrations of ethanol (**b**) (control used 50% DMSO; samples were 10 μg/mL in 50% DMSO); and Luteolin-7-O-diglucuronide (L7g) (**c**). Bars with different letters show significant differences between groups (*p* < 0.05) determined with the analysis of variance and Dunnett’s multiple comparison test.

Luteolin-7-O-diglucuronide had the same retention time (14.48 min) as the major compounds in the *P. frutescens* (L.) Britt. leaf extract, and when scanned using the UV pattern, these two peaks exhibited the same UV spectrum (Figure 1). This result was similar to that of a previous report by Fan [19]. Therefore, the major compound with retention time 14.48 min in the *P. frutescens* (L.) Britt. leaf extract was Luteolin-7-O-diglucuronide. As shown in Figure 2, *P. frutescens* (L.) Britt. leaf extract and Luteolin-7-O-diglucuronide had a good effect in terms of lowering cholesterol, so we used Luteolin-7-O-diglucuronide as the indicator component to optimize the extract solution.

According to the International Conference on Harmonization guidelines ICH Q2, we developed an HPLC method for the quantitative analysis of Luteolin-7-O-diglucuronide in *P. frutescens* (L.) Britt. leaf extract. Linear regression analysis for Luteolin-7-O-diglucuronide was performed by plotting the peak area (y) against the concentration (x, μg/mL) of Luteolin-7-O-diglucuronide standard solutions (Table 1). To assess the performance of the proposed method, analytical parameters were measured (Table 1). A satisfactory linearity was obtained in the range of 0.98–980 μg/mL with a determination coefficient of 0.999. The limit of quantification (LOQ) and limit of detection (LOD) for Luteolin-7-O-diglucuronide were determined to be three and ten times the signal-to-noise ratio, respectively. On the basis of these calculations, the LOD for Luteolin-7-O-diglucuronide was 6 μg/mL, whereas the LOQ was 17 μg/mL, indicating that the analytical method was acceptable with sufficient sensitivity. The relative standard deviation (RSD) values of the peak area of Luteolin-7-O-diglucuronide were 0.99–2.96% (intraday) and 2.03–2.74% (interday), which indicated that the precision of the instruments was good. To further validate the developed method, the spiked recoveries (30, 40, and 50 μg/mL) for the *P. frutescens* (L.) Britt. leaf extract ranged from 89.66 to 99.50%, and the RSD values were 0.77–4.62%, which indicated that the recovery of the method was good (Table 2). These parameters indicated that the HPLC method developed in this study has good precision, stability, repeatability, and accuracy and that it can be used to evaluate the Luteolin-7-O-diglucuronide content in *P. frutescens* (L.) Britt. leaves in different regions of China and Republic of Korea.

### 2.2. Selection of Origin of Perilla frutescens (L.) Britt. Leaves and Optimization of Extraction Conditions

*P. frutescens* (L.) Britt. leaves are mainly produced in China, Japan, North Korea, and South Korea. Due to the epidemic, we only collected 14 types of *P. frutescens* (L.) Britt. leaves from different regions of China (2 types) and Republic of Korea (12 types). After drying, all *P. frutescens* (L.) Britt. leaves were extracted using the maceration method. The extraction yield (all components; g/kg) and Luteolin-7-O-diglucuronide content (mg/g in *P. frutescens* (L.) Britt. leaves; dry weight) are shown in Table 3. The extraction yield of *P. frutescens* (L.) Britt. leaves from different regions and the Luteolin-7-O-diglucuronide content in the extract ranged from 23.6 to 60.9 g/kg and 1.10 to 29.73 mg/g, respectively. The highest extraction yield (60.9 g/kg) was observed in *P. frutescens* (L.) Britt. leaves from Hebei Province, China. According to the highest extraction yield (g/kg) of the final product and the cost and continuous supply of *P. frutescens* (L.) Britt. leaves, leaves from Hebei Province, China were chosen for subsequent experiments. 

Studies have shown that the total flavonoid extract of *P. frutescens*, which mainly contains apigenin and luteolin, inhibited hyperlipidemia in rats fed with a high-fat diet [10]. However, the main ingredient capable of lowering cholesterol was ignored. Our research showed that Luteolin-7-O-diglucuronide was the main compound in *P. frutescens* (L.) Britt. leaf extract, so we chose Luteolin-7-O-diglucuronide as the index component to optimize the extraction conditions of *P. frutescens* (L.) Britt. leaves. As established knowledge dictates, solvent selection, extraction temperature, and extraction time affected the yield of extraction processes. Nevertheless, it was imperative to underscore that the solvent’s inherent characteristics loom as the predominant determinants influencing extraction efficiency, owing to the proclivity of secondary metabolites within plant materials to be preferentially extracted by solvents possessing congruent chemical attributes [21]. Considering the need for the development of functional foods, we opted to extract *P. frutescens* (L.) Britt. leaves using an ethanol/water solution which was less toxic to the human body as the extraction solvent. 

In our study, we used water as a solvent with different concentrations of ethanol (0–100%) to obtain *P. frutescens* (L.) Britt. leaf extract. We calculated the extraction yield (all components; g/kg) and Luteolin-7-O-diglucuronide content (mg/g in *P. frutescens* (L.) Britt. leaves; Table 4) obtained using solvents with varying ethanol concentrations. The extraction yield (g/kg) was 24.0–62.7 g/kg, whereas the Luteolin-7-O-diglucuronide content was 0.013–0.548 mg/g. When the extraction solvent was 30% ethanol, *P. frutescens* (L.) Britt. leaf extract obtained the maximum extraction yield (62.7 g/kg) and the maximum Luteolin-7-O-diglucuronide content (0.548 mg/g). As the ethanol concentration increased, the solvent polarity decreased. Solvents with ethanol proportions less than 30% exhibited stronger polarity, whereas those with ethanol proportions greater than 30% exhibited weaker polarity. As reported in the literature, flavonoids and phenolic compounds were easily extracted from highly polar solvents [11,22]. To obtain a higher Luteolin-7-O-diglucuronide content and more flavonoid active ingredients, the cholesterol-lowering effect of the extract of *P. frutescens* (L.) Britt. leaves in 30% ethanol was investigated.

### 2.3. Cholesterol-Lowering Effects of Luteolin-7-O-diglucuronide and P. frutescens (L.) Britt. Leaf Extract

Figure 2a presents the cholesterol-lowering effects of *P. frutescens* (L.) Britt. leaf extracts from different regions of China and Republic of Korea. Compared with the control group, the groups treated with *P. frutescens* (L.) Britt. leaf extracts exhibited statistically significant activity in terms of displaying a cholesterol-lowering effect; however, no significant difference was observed in the cholesterol-lowering effects of extracts from different countries or regions. Figure 2b shows the cholesterol-lowering effects of *P. frutescens* (L.) Britt. leaf extracts obtained using solvents with different ethanol concentrations. Compared with the cholesterol-lowering effect in the control group, the cholesterol-lowering effects of *P. frutescens* (L.) Britt. leaf extracts increased as the ethanol concentration increased, and the difference was statistically significant. The *P. frutescens* (L.) Britt. leaf extract showed a significant cholesterol-lowering effect compared with the control treatment. With the increase in ethanol concentration (%), the cholesterol-lowering effect of *P. frutescens* (L.) Britt. leaf extracts was significantly enhanced and entered a plateau at 50% ethanol. Figure 2c shows the cholesterol-lowering effects of different concentrations of Luteolin-7-O-diglucuronide. Compared with the control treatment, Luteolin-7-O-diglucuronide exhibited a statistically significant cholesterol-lowering effect. The cholesterol-lowering effect of Luteolin-7-O-diglucuronide was dose-dependent.

Although Luteolin-7-O-diglucuronide exhibited good activity in terms of lowering cholesterol, Luteolin-7-O-diglucuronide was not the only compound in *P. frutescens* (L.) Britt. leaf extract that exhibited cholesterol-lowering effects, since the Luteolin-7-O-diglucuronide content in *P. frutescens* (L.) Britt. leaf extracts and the cholesterol-lowering effect did not show the same trend. Starting from 70% ethanol extract, the content of Luteolin-7-O-diglucuronide in *P. frutescens* (L.) Britt. leaves decreased, but their cholesterol-lowering effect did not change significantly. This result showed that there are other components in *P. frutescens* (L.) Britt. leaves that have a cholesterol-lowering effect. In our prior investigations, we identified eleven compounds in *P. frutescens* (L.) Britt. leaf extract, specifically protocatechuic acid, chlorogenic acid, caffeic acid, 4-methoxycinnamic acid, oleanolic acid, kaempferol-3-O-rutinoside, rosmarinic acid, luteolin, methyl-rosmarinic acid, apigenin, and 4′,5,7-trimethoxyflavone [23]. Notably, protocatechuic acid, chlorogenic acid, and caffeic acid have demonstrated their cholesterol-lowering effects through the inhibition of HMG-CoA reductase [24,25,26]. In the study conducted by Feng et al., it was substantiated that *P. frutescens* (L.) Britt. leaves contain apigenin and its analogues. Administration of oral dosages ranging from 50 to 200 mg/kg demonstrated their capacity to mitigate blood lipid levels and lipid accumulation within adipose tissues in experimental rodents. Moreover, it manifested the inhibition of the formation of lipid peroxidation products, amelioration of disturbances in lipoprotein metabolism, enhancement of antioxidant enzyme activity, and attenuation of hyperlipidemia incidence [18]. While previous investigations have established the cholesterol-lowering properties of apigenin, chlorogenic acid, and caffeic acid in *P. frutescens* (L.) Britt. leaves, it is noteworthy that Luteolin-7-O-diglucuronide is the main compound in *P. frutescens* (L.) Britt. leaf extract, and it has a good cholesterol-lowering effect. We continue to investigate the cholesterol-lowering effect mechanism of Luteolin-7-O-diglucuronide and *P. frutescens* (L.) Britt. leaf extract in this study.

**Figure 3:**
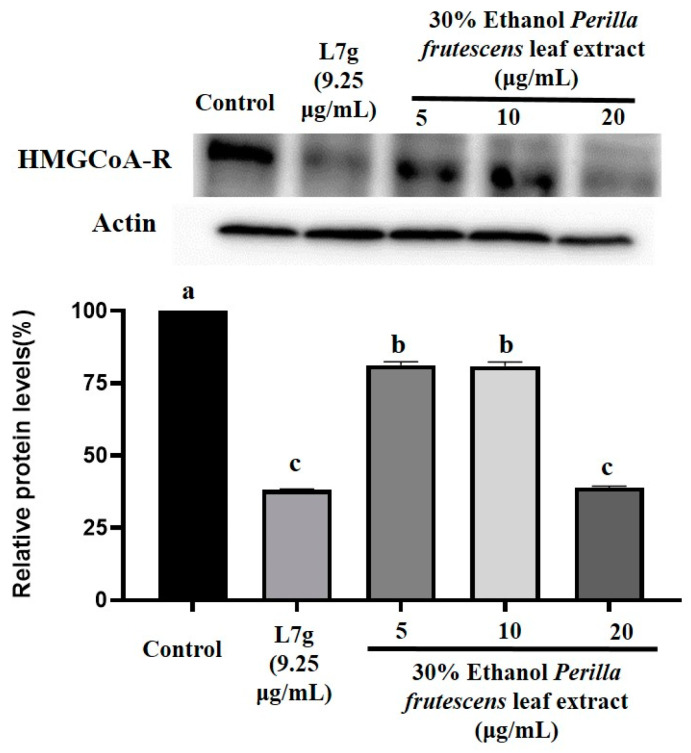
Effects of *P. frutescens* (L.) Britt. leaf extract (30% ethanol) from Hebei Province, China and Luteolin-7-O-diglucuronide (L7g) on lowering HMG-CoA reductase content in HepG2 cells. Control used 50% DMSO. Bars with different letters show significant differences between groups (*p* < 0.05) determined with analysis of variance and Dunnett’s multiple comparison test.

The liver is widely recognized as the primary organ responsible for cholesterol synthesis [27]. HMG-CoA reductase plays a crucial role in the synthesis of cholesterol in the liver [28]. Additionally, HepG2 cells primarily regulate the expression of HMG-CoA reductase and hepatic glycerolipid lipase [29]. Consequently, HepG2 cells can be used to assess the effectiveness of cholesterol-lowering treatments. In recent years, HepG2 cells have been widely used by researchers to assess cholesterol-lowering effects. For instance, Shuming Kou et al. employed HepG2 cells and high-cholesterol hamsters to investigate the synergistic cholesterol-lowering effect of five major alkaloids [30]. Similarly, Yunying Huang et al. utilized HepG2 cells to explore the cholesterol-lowering mechanism of bergamot extract [31]. To better understand the cholesterol-lowering mechanism of *P. frutescens* (L.) Britt. leaf extract and Luteolin-7-O-diglucuronide, we evaluated the expression of HMG-CoA reductase (Figure 3). Compared with the control group treatment, Luteolin-7-O-diglucuronide and the *P. frutescens* (L.) Britt. leaf extract significantly downregulated the expression of HMG-CoA reductase, and the difference was statistically significant. Moreover, when the concentration of *P. frutescens* (L.) Britt. leaf extract was 20 μg/mL, its downregulation effect on HMG-CoA reductase expression was equivalent to that of Luteolin-7-O-diglucuronide (9.25 μg/mL), with no statistical difference. *P. frutescens* (L.) Britt. leaf extract and Luteolin-7-O-diglucuronide significantly downregulated the expression of HMG-CoA reductase in HepG2 cells. Mansoureh Tavan’s research showed that after L929 cells were treated with different concentrations of *P. frutescens* (L.) Britt. leaf water extract (37.5–600 μg/mL), the cell survival rate was still greater than 90%, which indicated that *P. frutescens* (L.) Britt. leaf water extract had no toxicity to normal cells [32]. Puchadapirom’s research showed that after V79 cells were treated with different concentrations of *P. frutescens* (L.) Britt. leaf extract (100–250 μg/mL), the cell survival rate was still greater than 90% [33]. Lapatrada Mungmai’s results demonstrated the straightforward antimelanogenic effects of *P. frutescens* (L.) Britt. leaf extract at the optimum concentration (1.25–40 μg/mL) on B16F10 cells without inducing cytotoxicity or death of cells [34]. According to current research, there are currently no studies showing the cytotoxicity of *P. frutescens* (L.) Britt. leaf extract. Therefore, we concluded that *P. frutescens* (L.) Britt. leaf extract and Luteolin-7-O-diglucuronide could reduce cholesterol levels by downregulating the expression of HMG-CoA reductase. *P. frutescens* (L.) Britt. leaves have the potential to be developed as a functional food with a cholesterol-lowering effect. This study provides a theoretical basis for the further development of *P. frutescens* (L.) Britt. leaves.

## 3. Materials and Methods

### 3.1. Materials and Reagents

Cell lysis buffer, chemiluminescence kit, fetal bovine serum, 3-hydroxy-3-methyl-glutaryl-coenzyme A reductase (HMG-CoA reductase) antibody, Luteolin-7-O-diglucuronide, L-glutamine, NP-40, penicillin-streptomycin, phosphate-buffered saline with 0.05% TWEEN^®^ 20, pH 7.4 (PBST), SDS polyacrylamide, trifluoroacetic acid, and trypsin were purchased from Sigma-Aldrich Chemical Co. (St. Louis, MI, USA). HepG2 (human hepatocellular carcinoma) cells were purchased from American Type Culture Collection. The EZ Total Cholesterol Assay Kit was purchased from Abcam Co. (Cambridge, UK). Acetonitrile, chloroform, ethanol, and isopropanol were purchased from J. T. Baker Co. (Phillipsburg, NJ, USA). Ultrapure water used in this study was obtained from a Milli-Q water purification system from Millipore Co. (Bedford, MA, USA).

*P. frutescens* (L.) Britt. leaves were collected from Goesan-gun, Chungcheongbuk-do, Jecheon-si, Chungcheongbuk-do, Taean-gun, Chungcheongnam-do, Haenam-gun, Jeollanam-do, Gokseong-gun, Jeollanam-do, Jangsu-gun, Jeollabuk-do, Yeongcheon-si, Gyeongsangbuk-do, Gapyeong-gun, Gyeonggi-do, Uiseong-gun, Gyeongsangbuk-do, Nonsan-si, Chungcheongnam-do, Namwon-si, Jeollabuk-do, Jangheung-gun, and Jeollanam-do in Republic of Korea and Hebei Province and Guangdong Province in China in September 2022. The specimens were authenticated by Emeritus Professor H. J. Chi, Seoul National University, Republic of Korea. Dried *P. frutescens* (L.) Britt. leaves (L-2022-PF1-14) and voucher sample (RIC-2012-5) were stored at the Center for Efficacy Assessment and Development of Functional Foods and Drugs (Room 8510) at Hallym University.

### 3.2. Preparation of Perilla frutescens Leaf Extract

Dried *P. frutescens* (L.) Britt. leaves (1 g) from different regions (China and Republic of Korea) were successively extracted three times with 20 mL of 30% ethanol at 70 °C for 7 h using the maceration method. Dried *P. frutescens* (L.) Britt. leaves (1 g) from Hebei Province, China were extracted in 20 mL of different concentrations of ethanol at 70 °C for 7 h. The extract solution was filtered with filter paper and evaporated to dryness via rotary evaporation at 37 °C. Every sample was extracted three times. The yield was calculated using the following formula:

Extract yield (g/kg) = extract weight (g)/sample weight (g) × 1000

### 3.3. HPLC Analysis of Luteolin-7-O-diglucuronide

The *P. frutescens* (L.) Britt. leaf extract (2 mg) was dissolved in methanol to a concentration of 2 mg/mL and filtered through a 0.2 μm polyvinyl difluoride (PVDF) syringe filter. Luteolin-7-O-diglucuronide was prepared at 1 μg/mL in methanol. Agilent 1100 series HPLC/UV–Vis/MSD (Santa Clara, CA, USA) was used for analyzing the samples and standard solutions. The HPLC system was equipped with an auto-degasser, quaternary pump, autosampler, column thermostat, and diode array detector (DAD). The HPLC mobile phases used were acidic water (0.1% trifluoroacetic acid (95%) + acetonitrile (5%); (A) and acetonitrile (95%) + 0.1% trifluoroacetic acid (5%) (B). The *P. frutescens* (L.) Britt. leaf extract and Luteolin-7-O-diglucuronide solutions were analyzed at 254 nm and separated with a flow rate of 0.7 mL/min using a CAPCELL PAK DD Type C8 column (250 × 4.6 mm, 5 μm). The separation process was as follows: 0–15% B for 0–5 min, 15–25% B for 5–20 min, 25–100% B for 20–30 min, and 100% B for 30–35 min. The spectra of Luteolin-7-O-diglucuronide were analyzed at a working wavelength range of 190–400 nm.

### 3.4. Method Validation

To evaluate the quality of the analytical method, validation studies were performed using the optimized HPLC method according to the International Conference on Harmonization guidelines ICH Q2 (R2) [35]. The method was validated for precision, stability, repeatability, accuracy, LOQ, LOD, and calibration curves of Luteolin-7-O-diglucuronide.

### 3.5. Cell Culture

HepG2 cells were grown in Dulbecco’s modified Eagle’s medium supplemented with 10% fetal bovine serum, 1% penicillin–streptomycin, and 4 mM L-glutamine. The cells were cultured at 37 °C in a humidified atmosphere of 95% air to 5% CO_2_ [31].

### 3.6. Cellular Cholesterol Content Analysis

HepG2 cells were seeded in 6-well plates and incubated in Dulbecco’s modified Eagle’s medium in the absence or presence of *P. frutescens* (L.) Britt. leaf extract (different regions and different ethanol concentration extracts from Hebei Province, China; 10 μg/mL) and different concentrations of Luteolin-7-O-diglucuronide (0.46, 4.62, and 46.2 μg/mL) for 24 h. Cholesterol was measured using the EZ Total Cholesterol Assay Kit according to the manufacturer’s instructions. The cells were isolated using trypsin, washed with PBS, and centrifuged. Subsequently, 200 μL of chloroform: isopropanol: NP-40 (7:11:0.1) solution was added to the cell pellet, and the cells were homogenized on ice before being centrifuged at 15,000× *g* for 10 min in a microcentrifuge. The liquid (organic phase) was transferred to another tube, leaving the pellet, and dried at 50 °C to remove the chloroform. The samples were then vacuum-dried for 30 min to remove any trace organic solvents. Next, 200 μL of cholesterol assay buffer was added to the dried lipid and dissolved with sonication until the solution became turbid. The sample was subsequently transferred to a 96-well plate, and the absorbance was measured at 570 nm. All determinations were performed via replicate experiments with triplicate analysis.

### 3.7. Western Blotting

HepG2 cells were seeded in 6-well plates and incubated in Dulbecco’s modified Eagle’s medium in the absence or presence of different concentrations (5, 10, and 20 μg/mL) of *P. frutescens* (L.) Britt. leaf extract from Hebei Province, China and Luteolin-7-O-diglucuronide (9.25 μg/mL) for 24 h, and Western blot analysis was performed on the cells. Luteolin-7-O-diglucuronide was used as the positive control, and the control group remained untreated. The cells were separated using trypsin, washed with PBS, and centrifuged. The cell pellet was then treated with a cell lysis buffer (50 mM Tris-HCl, pH 7.4; 150 mM NaCl; 1 mM EDTA; 0.25% sodium deoxycholate; and 1% NP-40; supplemented with a protease inhibitor cocktail) to lyse the cells. After centrifugation, the cell extracts were prepared. The protein extract (25 μg) was separated via electrophoresis on an SDS polyacrylamide gel. The proteins in the gel were then electrotransferred to an immunoblot PVDF membrane, which was subsequently incubated with HMG-CoA reductase antibody and washed with PBST. Horseradish peroxidase-conjugated secondary antibodies were added and incubated before washing, and the protein bands were removed. Finally, the bands were visualized using an enhanced chemiluminescence kit. Band intensity was quantified using ImageJ software (https://ij.imjoy.io/, accessed on 7 October 2023) to determine the protein concentration of HMG-CoA reductase. All determinations were performed via replicate experiments with triplicate analysis.

### 3.8. Statistical Analysis

All data are expressed as the mean values ± standard deviation (SD). Differences between groups were compared using the Statistical Package for Social Science (SPSS 25.0) with one-way analysis of variance, and post hoc comparisons were evaluated using Dunnett’s test. All statistical tests were two-sided, and the significance level was set at *p* < 0.05.

## 4. Conclusions

In this study, the source of *P. frutescens* (L.) Britt. leaves, that is, Hebei Province, China, was selected on the basis of the extraction yield (%), and 30% ethanol was selected as the solvent for the extraction of *P. frutescens* (L.) Britt. leaves on the basis of the content of Luteolin-7-O-diglucuronide (mg/g in *P. frutescens* (L.) Britt. leaves) as the indicator component. The cholesterol-lowering effects of *P. frutescens* (L.) Britt. leaf extract and Luteolin-7-O-diglucuronide were evaluated, and the results show that the 30% ethanol extract was effective in reducing cholesterol levels by downregulating the expression of HMG-CoA reductase. This suggests that *P. frutescens* (L.) Britt leaves have significant health benefits and can be explored as a potentially promising food additive for the prevention of hypercholesterolemia-related diseases.

## Figures and Tables

**Table 1:** Analytical performances of the developed method for Luteolin-7-O-diglucuronide from *Perilla frutescens* (L.) Britt. leaf extract.

Analyte	Linearity (μg/mL)	Regression Equation	R^2^	LOD (μg/mL)	LOQ (μg/mL)	RSD (%)					
						2.5 mg/mL		5.0 mg/mL		10 mg/mL	
						Intra	Inter	Intra	Inter	Intra	Inter
Luteolin-7-O-diglucuronide	0.98–980	Y = 22,850 X + 185.59	0.999	6	17	0.99	2.03	2.96	2.39	1.54	2.74

**Table 2:** Recovery of Luteolin-7-O-diglucuronide using the developed method for the spiked *P. frutescens* (L.) Britt. leaf extract.

Analyte	30 μg/mL		40 μg/mL		50 μg/mL	
	Recovery (%)	RSD (%)	Recovery (%)	RSD (%)	Recovery (%)	RSD (%)
Luteolin-7-O-diglucuronide	89.66	4.62	94.79	3.05	99.50	0.77

**Table 3:** Extract yield (%) and Luteolin-7-O-diglucuronide (mg/g) content in *P. frutescens* (L.) Britt. leaves from different regions in China and Republic of Korea.

Sample No.	Location	Extract Yield (g/kg)	Luteolin-7-O-diglucuronide (mg/g in *P. frutescens* (L.) Britt. Leaves)
1	Goesan-gun, Chungcheongbuk-do, Republic of Korea	31.7	0.539 ± 0.001
2	Jecheon-si, Chungcheongbuk-do, Republic of Korea	34.2	0.549 ± 0.005
3	Taean-gun, Chungcheongnam-do, Republic of Korea	34.5	0.325 ± 0.001
4	Haenam-gun, Jeollanam-do, Republic of Korea	36.2	0.689 ± 0.008
5	Gokseong-gun, Jeollanam-do, Republic of Korea	35.2	0.285 ± 0.003
6	Jangsu-gun, Jeollabuk-do, Republic of Korea	47.2	0.391 ± 0.026
7	Yeongcheon-si, Gyeongsangbuk-do, Republic of Korea	26.3	0.406 ± 0.013
8	Gapyeong-gun, Gyeonggi-do, Republic of Korea	31.4	0.059 ± 0.026
9	Uiseong-gun, Gyeongsangbuk-do, Republic of Korea	29.7	0.758 ± 0.076
10	Nonsan-si, Chungcheongnam-do, Republic of Korea	44.9	1.335 ± 0.076
11	Namwon-si, Jeollabuk-do, Republic of Korea	22.8	0.127 ± 0.007
12	Jangheung-gun, Jeollanam-do, Republic of Korea	27.6	0.293 ± 0.012
13	Anguo City, Hebei Province, China	60.9	0.445 ± 0.001
14	Guangzhou City, Guangdong Province, China	23.6	0.026 ± 0.001

**Table 4:** Luteolin-7-O-diglucuronide (mg/g) content in *P. frutescens* (L.) Britt. leaves extracted using different ethanol concentrations.

Sample No.	Ethanol Concentration (%)	Extract Yield (g/kg)	Luteolin-7-O-diglucuronide (mg/g in *P. frutescens* (L.) Britt. Leaves)
1	0	47.1	0.171 ± 0.017
2	10	62.1	0.154 ± 0.006
3	20	59.9	0.250 ± 0.005
4	30	62.7	0.548 ± 0.009
5	40	57.1	0.548 ± 0.005
6	50	59.4	0.540 ± 0.016
7	60	56.9	0.513 ± 0.016
8	70	56.3	0.251 ± 0.028
9	80	52.2	0.046 ± 0.020
10	90	35.2	0.017 ± 0.001
11	100	24.0	0.013 ± 0.001

## Data Availability

Not applicable.

## Sample Availability

All the samples are available from the authors.
