# Correction: Wu et al. Determination of Luteolin-7-O-diglucuronide in *Perilla frutescens* (L.) Britt. Leaf Extracts from Different Regions of China and Republic of Korea and Its Cholesterol-Lowering Effect. *Molecules* 2023, *28*, 7007

**DOI:** 10.3390/molecules30224438

**Published:** 2025-11-17

**Authors:** Zhaoyang Wu, Sangyoun Lee, Beomgoo Kang, Sookyeong Lee, Kyochul Koo, Jaeyong Lee, Soonsung Lim

**Affiliations:** 1 Department of Food Science and Nutrition, Hallym University, 1 Hallymdeahak-gil, Chuncheon 24252, Republic of Korea; wzy19970202@163.com (Z.W.); ilove0977@nate.com (S.L.); 2 Institute for Liver and Digestive Diseases, Hallym University, 1 Hallymdeahak-gil, Chuncheon 24252, Republic of Korea; briansylee@naver.com; 3 Department of Biochemistry, College of Medicine, Hallym University, 1 Hallymdeahak-gil, Chuncheon 24252, Republic of Korea; kbgda87@naver.com (B.K.); jyl3746@gmail.com (J.L.); 4 Institute of Korean Nutrition, Hallym University, 1 Hallymdeahak-gil, Chuncheon 24252, Republic of Korea; 5 COSFarm Co., Ltd., Corporate Research Institute, 3F 162, Saeteo-gil, Seonggeo-eup, Seobuk-gu, Cheonan-si 12446, Republic of Korea; rnrycjf76@naver.com

I am writing to inform you of an error in my recently published paper titled “Determination of Luteolin-7-O-diglucuronide in *Perilla frutescens* (L.) Britt. Leaf Extracts from Different Regions of China and Republic of Korea and Its Cholesterol-Lowering Effect” (https://doi.org/10.3390/molecules28207007).

Upon further review, I noticed the following errors: In this article, we misspelled the name of the target compound (Luteolin 7-Glucuronide).

The authors have made the following revisions to this article [1]:

“It must be Luteolin-7-O-diglucuronide, and we would like to correct all misspelled words in the article including Figure 1.”

**Figure 1:**
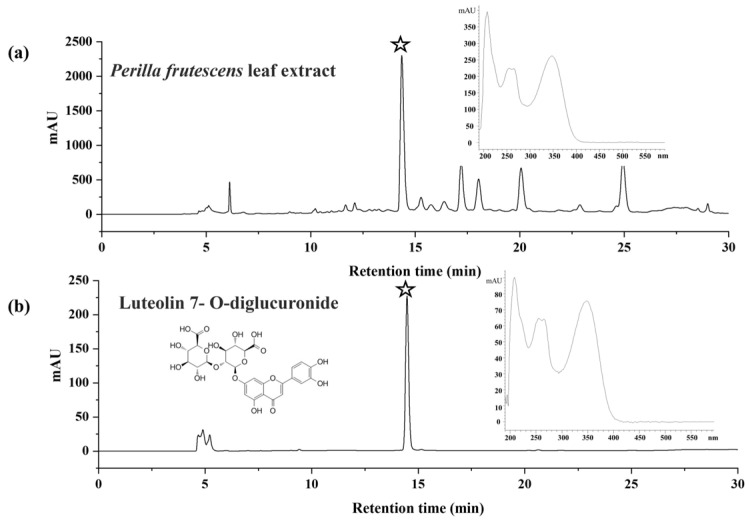
HPLC spectra of 30% ethanol extract of *P. frutescens* (L.) Britt. leaves from Hebei Province, China (**a**) and Luteolin-7-O-diglucuronide (**b**), stars in the figure are the target compound in the 30% ethanol extract of *P. frutescens* (L.) Britt. leaves.

The authors state that the scientific conclusions are unaffected. This correction was approved by the Academic Editor. The original publication has also been updated.
